# DNA Nanotechnology in the Undergraduate Laboratory: Toehold-Less Strand Displacement in Switchback DNA

**DOI:** 10.1021/jacsau.4c01204

**Published:** 2025-01-29

**Authors:** Bharath Raj Madhanagopal, Arun Richard Chandrasekaran

**Affiliations:** † The RNA Institute, University at Albany, State University of New York, Albany, New York 12222, United States; ‡ Department of Nanoscale Science and Engineering, University at Albany, State University of New York, Albany, New York 12222, United States

**Keywords:** Upper-division undergraduate, Biochemistry, Interdisciplinary/multidisciplinary, Hands-on learning/manipulatives, Electrophoresis, Molecular properties/structure, Nanotechnology, Nucleic acids/DNA/RNA, Undergraduate research, DNA nanotechnology

## Abstract

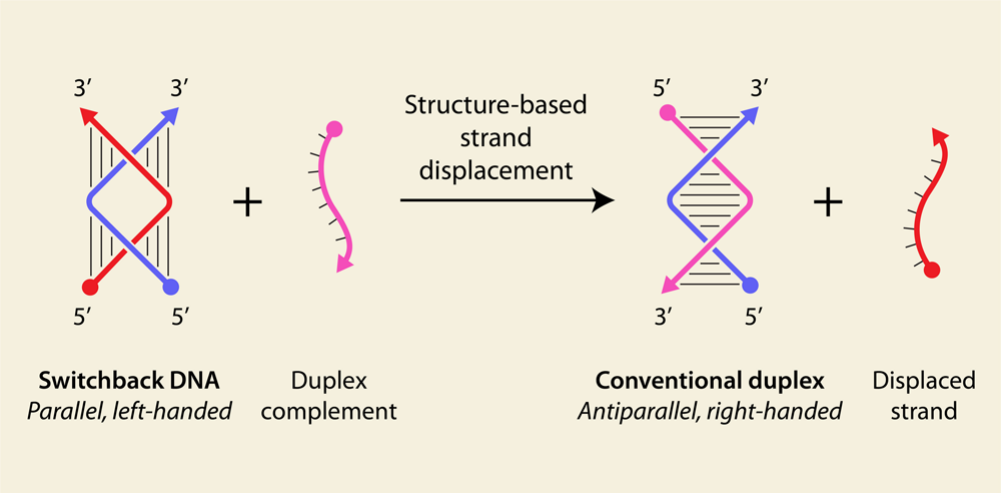

Dynamic DNA nanostructures that reconfigure into different shapes are used in several applications in biosensing, drug delivery, and data storage. One of the ways to produce such structural transformations is by a process called strand displacement. This laboratory experiment demonstrates a strand displacement reaction in a two-stranded DNA nanostructure called switchback DNA by the addition of a third strand. In this process, the difference in the affinity between the component DNA strands is used to convert switchback DNA into conventional duplex DNA. Students are introduced to the concept through gel electrophoresis and quantitative analysis of DNA nanostructure reconfiguration. The experiment presented here is an example of DNA nanotechnology-based exercises in an undergraduate setting and is tailored for adaptation in a chemistry, biology, or biochemistry laboratory with minimal costs.

## Introduction

DNA self-assembly has been used in the construction of a variety of nanostructures, such as polyhedral objects and multidimensional arrays.^1,2^ Assembly of these structures involves different types of DNA motifs, with examples including the DNA double crossover (DX) motif,^3^ triple crossover (TX) motif,^4^ paranemic crossover (PX) DNA motif,^5^ and multiarm DNA stars.^6,7^ Further, DNA nanostructures can be made to reconfigure based on external stimuli such as pH, ions, or other nucleic acids.^8^ While various strategies have been used to induce structural reconfiguration in nucleic acid nanostructures, toehold-mediated strand displacement is a widely used concept in dynamic DNA nanostructures for applications in biosensing, molecular computation, drug delivery, and materials science.^9,10^ As newer DNA motifs and nanostructures are being designed, modifications to displacement strategies have also been developed. For example, light-responsive linkers have been used to create caged toeholds,^11^ photocleavable linkers to control displacement reactions,^12^ and aptamers that respond to nucleic acids or proteins have been used for different functions.^13^ In contrast to toehold-mediated strand displacement, reconfiguration of DNA nanostructures can also be based on the affinity of component DNA strands to form different types of DNA motifs.^14^ In this educational article, we discuss the switchback motif^14,15^ and the concept of toehold-less strand displacement process from switchback DNA to conventional duplex DNA, with the results visualized by gel electrophoresis. The pedagogical value of this experiment is to introduce undergraduate students to a new type of DNA motif, combined with practical skill development in gel electrophoresis, an often-used technique in biophysics, molecular biology, and biochemistry for a range of investigations such as analyzing photoinduced oxidative DNA damage,^16^ quantifying DNAzyme activity,^17^ determining protein concentration,^18^ characterizing DNA-cleaving metal complexes,^19^ biostability of DNA motifs^20^ and analyzing the molecular topology of DNA nanoswitches.^21^

DNA nanostructures are typically characterized by methods such as atomic force microscopy, cryo-electron microscopy, transmission electron microscopy, scanning electron microscopy, dynamic light scattering, and size exclusion chromatography.^22,23^ While such advanced techniques allow high-resolution analyses of assembled structures, gel electrophoresis remains an easy-to-adapt and routine method for DNA nanostructure characterization. Recently, the analysis of DNA origami structures by gel electrophoresis has been developed for middle school, high school, and undergraduate laboratories.^24^ Our own undergraduate laboratory experiment protocols have described the use of gel electrophoresis for analyzing the molecular topology of DNA nanoswitches^21^ and for evaluating the biostability of DNA motifs.^20^ Several other articles and laboratory protocols have introduced various concepts of DNA nanotechnology to students.^17,25−28^ The current laboratory experiment adds to this series of demonstrations on DNA nanotechnology concepts in an undergraduate laboratory setting.

## Hazards

***CAUTION:** Acrylamide and tetramethylethylenediamine (TEMED) are hazardous chemicals. Acrylamide is a carcinogen, and exposure can occur via inhalation (if aerosolized), ingestion, and skin absorption. Care should be taken when using acrylamide to prepare gels. TEMED is a flammable liquid and must be handled with care and used only under a chemical fume hood. Avoid contact with skin or clothing and wear personal protective equipment/face protection while handling the chemicals to prevent accidental exposure. The electrophoresis apparatus and the power supply must be handled cautiously as they pose electrical hazards. To avoid electric shocks, students should use care when plugging the gel boxes into the power supply. Exercise caution while using the gel imager by wearing appropriate skin protection to avoid potential exposure to UV irradiation.*

## Concepts and Results

**Figure 1:**
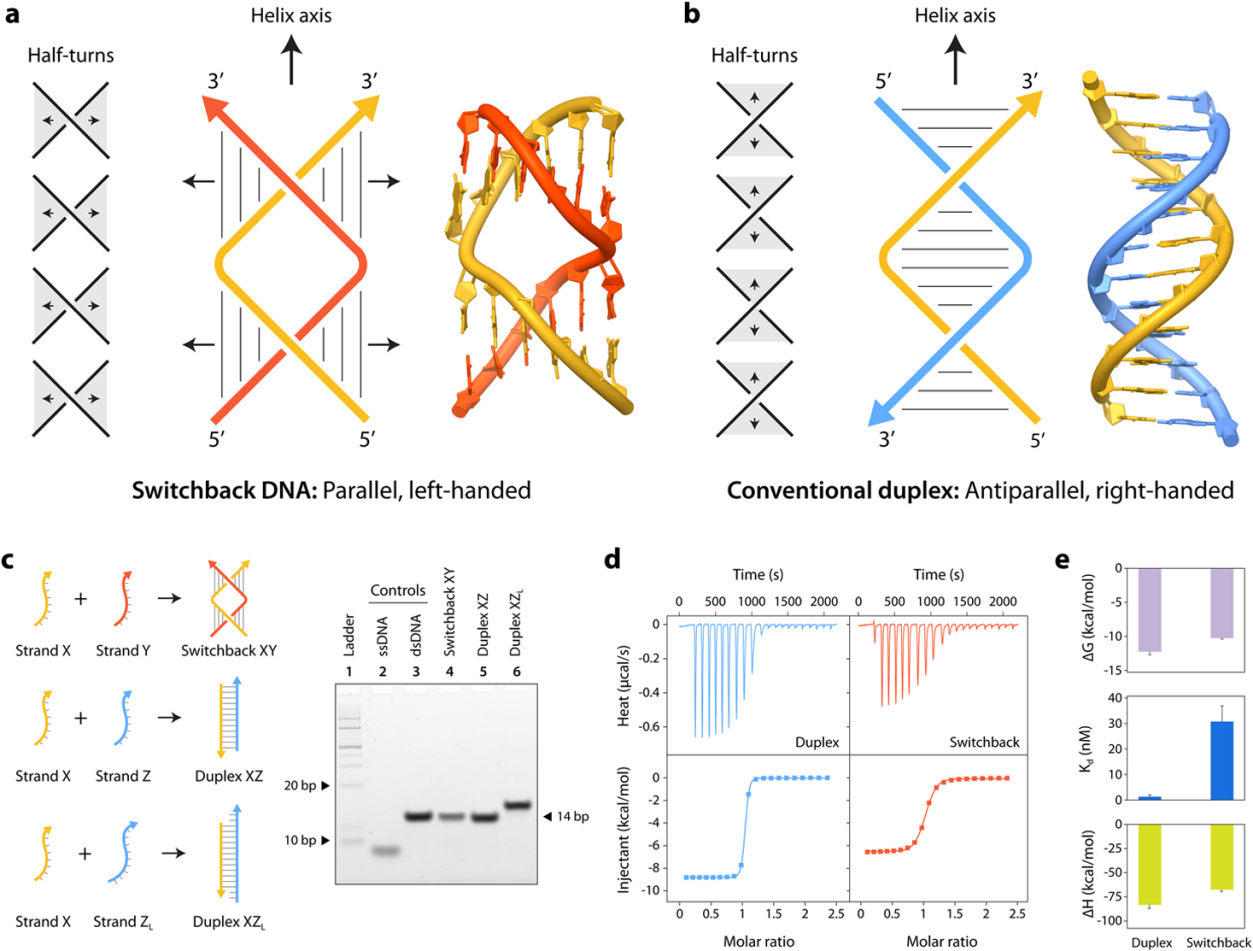
Switchback DNA and conventional duplex. Schematic and model of (a) switchback DNA and (b) conventional duplex. The models are adapted from PDB ID 8EPF(29) and PDB ID 1BNA,^30^ respectively. Arrows denote 3′ ends of DNA strands. The structure contains two half-turn domains, with each half-turn domain consisting of six base pairs. The switchback DNA structure has a global left-handed twist, with the helical axis of the full structure being perpendicular to the helical axis of the half-turn domains. The yellow and red strands are complementary in the switchback sense. (c) Nondenaturing polyacrylamide gel electrophoresis (PAGE) analysis of switchback DNA. (d) Isothermal titration calorimetry (ITC) thermograms of conventional duplex and switchback DNA. (e) Thermodynamic parameters of conventional duplex and switchback DNA. Adapted from ref (14). Available under a CC-BY 4.0 license. Copyright 2024 Madhanagopal, Talbot, and Rodriguez et al.

This undergraduate laboratory experiment is based on our recent study of switchback DNA, a motif assembled from two strands of DNA.^14^ In switchback DNA, units of six base pairs that constitute a B-DNA half-turn are aligned laterally in contrast to the coaxial arrangement of half-turns in the conventional double helical structure of DNA (Figure 1a).^14,29,31^ That is, the helical axis of the half-turns is perpendicular to the axis of the global helix. Because the strands switch back after each half-turn, the overall helix is left-handed with the strands running parallel to each other. In our previous study on switchback DNA, we assembled a heterodimeric switchback DNA using two strands X and Y (Table S1). To compare the properties of switchback DNA with that of a conventional duplex, we designed a strand (Z) that can pair with strand X to form a conventional duplex DNA. In this context, strands X and Y are complementary in the switchback sense, while strands X and Z are complementary in the conventional duplex sense. We validated the assembly of the switchback XY using nondenaturing polyacrylamide gel electrophoresis (PAGE) (Figure 1c). The formation of switchback DNA was confirmed by the fact that it migrated similar to the conventional duplex of the same length and the absence of bands corresponding to single strands. We also studied the thermodynamics of the formation of conventional duplex XZ and switchback DNA XY using isothermal titration calorimetry (ITC). Strands X, Y, and Z were 14 nt long, and the titrations were performed at 25 °C in 1× tris-acetate-EDTA (TAE) buffer (pH 8) containing 12.5 mM magnesium acetate. The ITC results of that study showed a Δ*G* of −12.23 kcal/mol for conventional duplex formation and a Δ*G* of −10.26 kcal/mol for switchback DNA formation (Figure 1d,e).^14^ The measured dissociation constants (*K*_d_) were 1.3 nM for the conventional duplex and 30.7 nM for the switchback DNA. Since the Δ*G* for the conventional duplex was more negative (by about 2 kcal/mol) than switchback DNA and the *K*_d_ values of the two structures differed by ∼23 times, the conventional duplex was considered thermodynamically more stable than its switchback counterpart. Analysis of the Δ*H* values also revealed that the enthalpy contribution to the stability of the structure is relatively less for switchback DNA than for conventional duplex although the two structures have the same number of complementary base pairs (Figure 1e). However, the entropic penalty for the formation of the conventional duplex was higher than for switchback DNA formation, which is perhaps a consequence of disruption in the stacked bases in switchback DNA. These differences in the thermodynamic stability of the two complexes manifested in the preference of strand X to bind to strand Z over strand Y. This led us to test whether a duplex complement (Z) can displace a switchback complement (Y) from an assembled switchback DNA, a process that would be toehold-less strand displacement.

**Figure 2:**
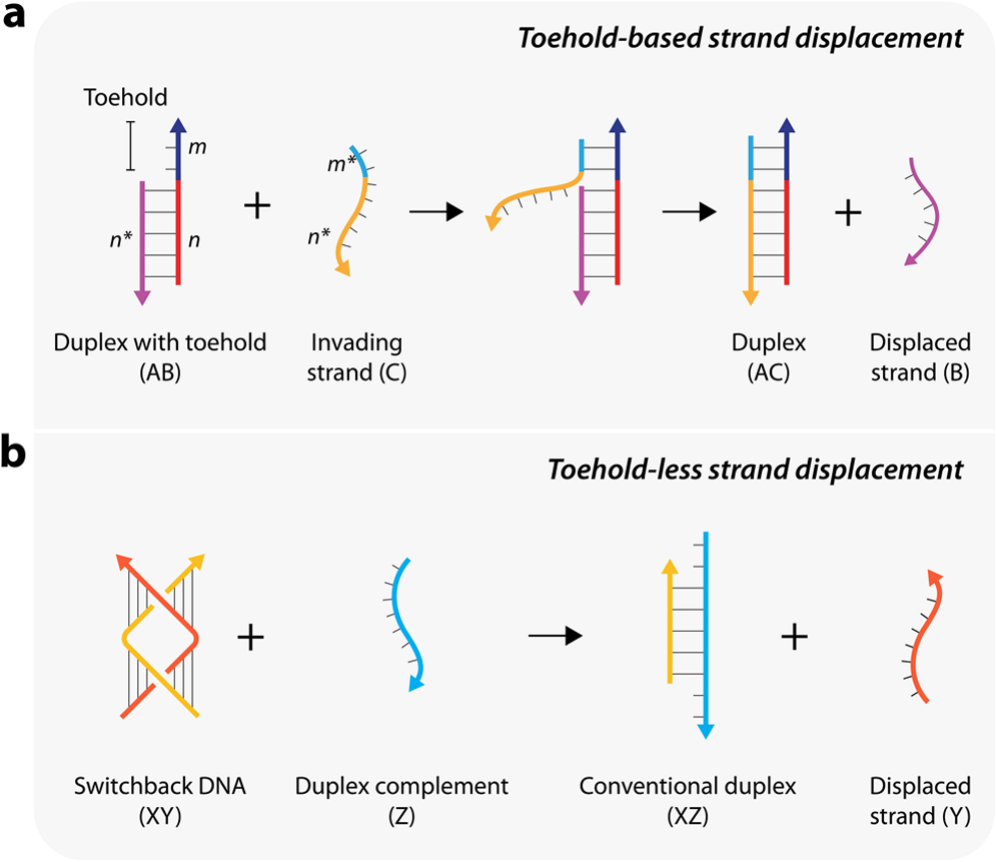
Strand displacement reactions. (a) In toehold-mediated strand displacement, strand C binds to the toehold region *m* in strand A and proceeds to displace strand B from duplex AB producing AC with a higher number of base pairs. (b) In toehold-less strand displacement, strand Z displaces strand Y from switchback DNA (XY) producing a conventional duplex (XZ).

In a typical toehold-mediated strand displacement reaction, an incoming single stranded DNA with a full sequence complementarity displaces a partially complementary strand from a prehybridized duplex (Figure 2a).^32^ The invading strand initially binds to a single-stranded region called a toehold, and this binding event triggers a branch migration process that results in the removal of the previously partially bound strand to produce a duplex with more base pairs than the original duplex. The concept of toehold-based strand displacement has been used often in DNA nanotechnology and is now well established.^9,10^ In the context of switchback DNA, a duplex complement displaces a switchback complement from a preassembled switchback DNA, resulting in the formation of a conventional duplex (Figure 2b). Unlike the toehold containing duplex in the toehold-mediated strand displacement strategy, no bases in the switchback DNA are unpaired, and there is no single-stranded region in the structure. Despite that, the displacement of the switchback complement strand is favored due to the higher thermodynamic stability of the conventional duplex (formed by the invading strand Z with the common strand X) than the initial switchback DNA XY. This toehold-less stand displacement strategy is based on the difference in the structural stability rather than the sequence-based affinity in toehold-based strand displacement and could be used in specific folding of DNA structures within a mixture,^33^ in controlling metastable DNA motors,^34^ and in tuning the biostability of DNA nanostructures by reconfiguration between structures containing different number of crossovers.^35^

**Figure 3:**
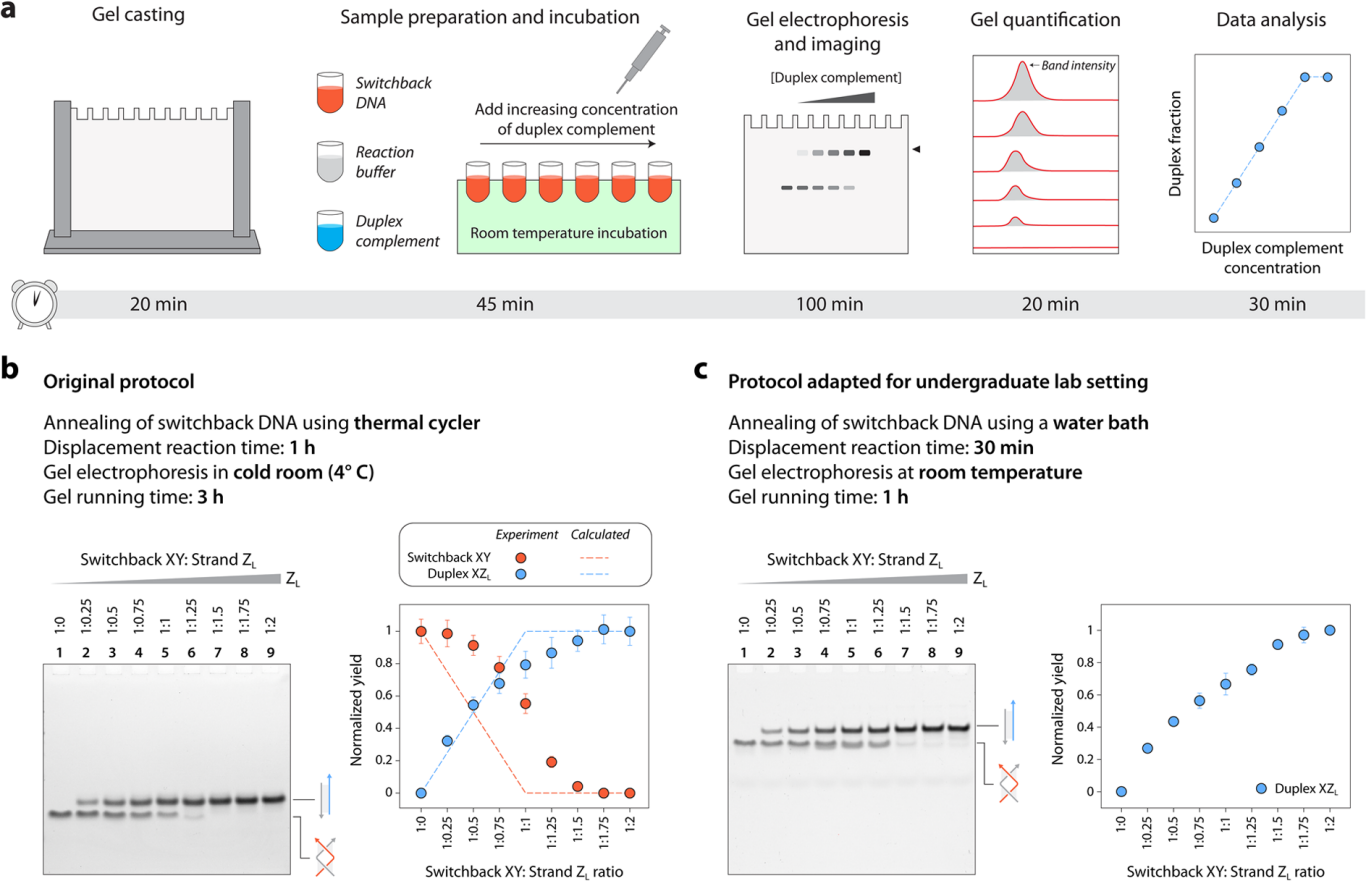
Protocol and expected results. (a) Nondenaturing polyacrylamide gel casting, sample preparation, loading incubated samples on a nondenaturing polyacrylamide gel and running the gel, and quantification of bands corresponding to duplex DNA in the gel image to estimate the yield of duplex product at various concentrations tested. The estimated time durations for each step are shown below the illustration. (b) Addition of the duplex complement to a preassembled switchback DNA causes displacement of one of the strands in the switchback DNA, resulting in duplex formation. (c) Experiment adapted for an undergraduate laboratory setting yielded results similar to previously published data shown in (b). Data in panel (b) is reproduced from ref (14). Available under a CC-BY 4.0 license. Copyright 2024 Madhanagopal, Talbot, and Rodriguez et al.

To perform the strand displacement reaction with switchback DNA in an undergraduate laboratory setting, here we provide an experimental workflow that can be completed in under 3 h (Figure 3a, Table S2). In this laboratory experiment, students analyze the reconfiguration of switchback DNA to conventional duplex DNA structures using non-denaturing PAGE. To identify the product and to discern it from the initial switchback structure, we used a longer duplex complement Z_L_ (same sequence as strand Z but with 2 additional Ts at each terminus). The strand displacement can be observed on a gel by the different migration of the start and end structures (Figure 1c, lane 6). The use of longer complement Z_L_ obviates the need to use more sophisticated techniques or expensive fluorophore-labeled strands and makes the experiment suitable for an undergraduate laboratory. In previous work, we annealed switchback DNA using a thermal annealing protocol that required a thermal cycler and performed nondenaturing PAGE in the cold room (4 °C) (Figure 3b). For adaptation in an undergraduate setting, we tested the assembly of switchback DNA using a hot water bath and modified the gel electrophoresis protocols to be performed at room temperature on the bench (∼20 °C) instead of a cold room. We confirmed that these changes did not affect the assembly and displacement process, and the results were similar to our original report^14^ (Figure 3c). The bands corresponding to the product duplex appeared with progressive intensities as the concentration of strand Z_L_ increased. The product band was well resolved from the substrate switchback band under the modified protocol, similar to the previously reported electrophoresis protocol. The intensity of the duplex band increased steadily reaching the maximum intensity at [strand X]:[strand Z_L_] molar ratio of 1:1.75, similar to our previous report.^14^

The structures can be assembled by the instructor using the protocol in Supplementary Note 1 and provided to students when they start the laboratory experiment. The in-lab experiment involves mixing the duplex complement at different ratios to the switchback DNA, incubation at room temperature, gel electrophoresis, and gel imaging (a separate step-by-step student hand-out is provided in the SI: Supplementary Note 2). Our experimental design suggests gel quantification and data analysis as postlab exercises but can be performed in the lab if timings are modified. In our own laboratory, our workflow included a demonstration of gel analysis and plotting to all the students together, followed by each student performing the analysis independently. The instructors could perform a control experiment beforehand for the students to compare their results with. As an alternative, we have provided the data set from our previously published research experiments^14^ (the data shown in Figure 3b) that can be used as a control data set to validate results (Table S3).

**Figure 4:**
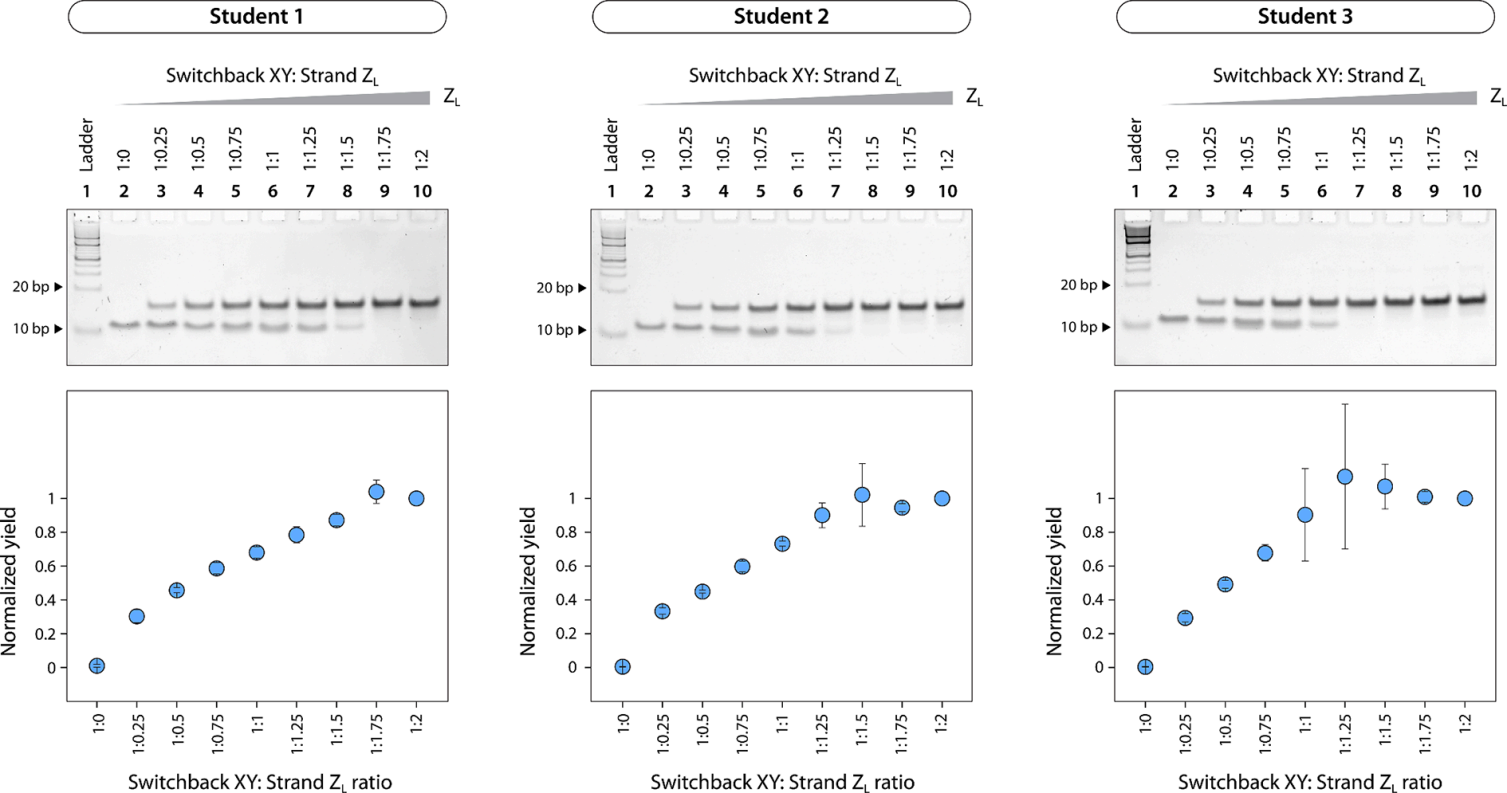
Representative results from experiments performed by undergraduate students. Students were given the manual in Supplementary Note 2 and were provided with annealed DNA complexes and reagents. Gel analysis was done using ImageLab and data plotted using Origin. Error bars are standard deviations from experiments performed in triplicates by each student.

Representative results of the experiments performed by undergraduate students in our laboratory are provided in Figure 4. Students were provided with the assembled switchback DNA motif, duplex complement DNA, and TAE buffer containing Mg^2+^ (10×), the buffer in which the structures are assembled (1×). Students performed the displacement assay at room temperature (on the bench), loaded the samples and ran them on an 18% nondenaturing polyacrylamide gel, stained the gels using GelRed, imaged the gels, quantified the bands, and analyzed the data. The plots created using data from the gels showed expected trends and were consistent between different students (Figure 4). Results obtained by the students were also similar to our previously published results.^14^

## Discussion

The field of DNA nanotechnology has recently expanded to several new concepts, necessitating the development of new curricula to introduce the field to students at early educational levels. The laboratory experiment presented here is one such example that introduces a new DNA motif and demonstrates strand displacement, a key tool used in DNA nanotechnology. The experiment is most suited for undergraduate students who have learnt basic pipetting and gel electrophoresis techniques, such as third-year undergraduate students who have taken chemistry, biology, biochemistry, molecular biology, or genetics laboratory courses and have performed gel electrophoresis. We would like to note that we sometimes have first year undergraduate students who get trained in these skill sets and are able to perform gel electrophoresis on their own in a research lab setting. In the student manual provided with this article (Supplementary Note 2), each student performs the strand displacement experiment in triplicates (so did undergraduates in our lab, data provided in Figure 4). Depending on the available resources and time constraints, the experiment can be performed in groups of three, with each student performing a replicate. The experiment uses equipment typically available in an undergraduate lab, and the DNA cost is minimal. The DNA strands required for this experiment can be ordered from companies such as Integrated DNA Technologies (IDT), and cost a total of ∼ $19 for all three strands when ordered at the 100 nmol scale. For the replicate experiment described here, we only use 0.33 nmol for each strand of DNA per student. The remaining DNA strands can be stored frozen for many years and be used for several courses. Typical gel electrophoresis setups can accommodate up to four gels at a time, which allows this experiment to be performed by 20–30 students using 6–8 gel setups if done in parallel. Gel electrophoresis modules including buffer tanks, gel casting stand, gel chamber and gel plates cost ∼ $900 and can be repeatedly used for several years. Recent advances and efforts toward 3D printing lab equipment^36^ can also reduce the initial investment cost for such experiments, making it more adaptable for low-resource settings. There are also portable imaging systems available for ∼ $100 that can be used with smartphones for image capture and analysis through phone-based applications, with several recent works focusing on the development of low-cost gel documentation systems.^37,38^ Our method already uses the nontoxic intercalating dye GelRed for gel staining, making this experiment student-safe and does not require special waste disposal procedures. An alternative to reducing the overall time of the experiment is the use of commercially available precast polyacrylamide gels, but that option would increase the overall cost of the experiment.

The usefulness of this laboratory experiment extends beyond improving the undergraduate curriculum. It allows students to understand scientific concepts in a new and emerging field of study and may motivate them to pursue research in related areas. Introducing the original research related to the experiments performed in the undergraduate laboratory will also help in enhancing student learning. Some key skills that this laboratory experiment provides students are pipetting skills, nucleic acid gel electrophoresis and imaging, gel image quantification, and replicate data analysis to calculate average and error values. The gel analysis and postlab exercises allow students to compare the two DNA structures, and can also help assess student learning in large undergraduate laboratories. The pedagogical goals achieved in this study are as follows: (1) Skill development–the students successfully prepared the samples using micropipettes and performed polyacrylamide gel electrophoresis in the stipulated time, (2) Data analysis–the students performed image analysis and quantified the bands on the gels successfully, (3) Conceptual understanding of DNA nanotechnology–the students presented their results and discussed their data in the group meeting, demonstrating their grasp of structural transformations in DNA nanostructures, (4) Statistical analysis–this experiment allowed the students to learn the importance of reproducibility in scientific experiments by performing simple statistical analysis. In research settings for undergraduate students such as ours, student evaluation is achieved through their presentations in weekly group meetings with constant feedback for improvement of laboratory skills, data analysis and interpretation, and accurate representation of data. As part of a series of laboratory experiments on DNA nanotechnology for undergraduate students,^20,21,24^ this experiment takes us a step closer to developing a collection of laboratory protocols for a DNA nanotechnology undergraduate laboratory course.

## Materials and Methods

### Oligonucleotides

All the DNA strands were purchased from Integrated DNA Technologies (IDT). The stock solutions of the DNA strands at a concentration of ∼100 μM were prepared by resuspending them in nuclease-free water. The exact concentrations were measured using a NanoDrop 2000 spectrophotometer using the molar extinction coefficients of the strands provided by IDT. All further dilutions were made in nuclease-free water. The sequences used in the study are shown in Table S1.

### Assembly of DNA Complexes

The DNA solutions were annealed in tris-acetate EDTA buffer containing 40 mM Tris base (pH 8.0), 20 mM acetic acid, 2 mM EDTA, and 12.5 mM magnesium acetate (1× TAE-Mg^2+^). Typically, we use a 10× buffer to prepare all the oligonucleotide samples. To prepare switchback DNA, the required volumes of the stock solution of strands X and Y were mixed with 10× TAE-Mg^2+^, and the final volume was adjusted with water. The solution was annealed by suspending the microfuge tubes containing the DNA solution in a water bath heated to 90 °C and cooled to room temperature over about 4 h. The solutions were stored at 4 °C. Single-stranded DNA strand Z_L_ was prepared in a final 1× TAE-Mg^2+^ buffer. A detailed step-by-step procedure for lab instructors is provided in **Supplementary Note 1**.

### Polyacrylamide Gel Electrophoresis (PAGE)

Nondenaturing gels containing 18% polyacrylamide (19:1 acrylamide/bis(acrylamide)) were run at room temperature (20 °C) at a constant voltage of 150 V constant voltage) in 1× TAE/Mg^2+^ running buffer. The gels were stained with GelRed by shaking the gels in a tray with 50 mL deionized water containing 2 μL GelRed for 10 min. After staining, the gels were destained in 50 mL deionized water for 5 min. The gels were imaged on a Bio-Rad Gel Doc XR+ imager using the default settings for GelRed with UV illumination. The exposure time was adjusted to ensure no saturated pixels in the bands of interest. The band intensities were quantified using Image Lab software (free to download online). We use the Mini-PROTEAN Tetra Vertical Electrophoresis system (BioRad), and optimized gel running conditions to be 150 V for 60 min. The voltage and running time might have to be optimized based on the type of gel electrophoresis equipment available in the laboratory.

## Supporting Information Available

Materials and methods, notes for instructors, student instruction manual, and worksheet for students (PDF)
