# Supplementary material for: DNA Nanotechnology in the Undergraduate Laboratory: Toehold-Less Strand Displacement in Switchback DNA
Source: JACS Au. 2025 Jan 29;5(2):1069–75. doi: 10.1021/jacsau.4c01204 (PMC11862946; doi:10.1021/jacsau.4c01204)
Supplement: Supplementary file 1 — au4c01204_si_001.pdf [file au4c01204_si_001.pdf]

Supporting Information

DNA nanotechnology in the undergraduate laboratory: Toehold-less strand displacement in switchback DNA

Bharath Raj Madhanagopal[1] and Arun Richard Chandrasekaran[1,2]*

[1]The RNA Institute, University at Albany, State University of New York, Albany, New York 12222, USA.

[2]Department of Nanoscale Science and Engineering, University at Albany, State University of New York, Albany, New York 12222, USA.

This PDF file includes:

1. Supporting Tables
2. Supplementary note 1: Reagent preparation for instructor
3. Supplementary note 2: Student instruction manual
4. Example student worksheet

Table S1. Sequences of DNA used in this experiment.

**Strand Name**	**Sequence (5′ to 3′)**	**Length**
X	TACCAGCCGAACCT	14 nt
Y	TGCTGGTGGTTCGT	14 nt
Z_L	TTTGGTTCGGCTGGTTTT	18 nt

Table S2. List of reagents used in this experiment.

**Reagent**	**Supplier**	**Catalog #**
Nuclease-free water	Invitrogen	10977-015
40% Polyacrylamide solution (19:1 acrylamide/bisacrylamide)	National Diagnostics	EC-850
GelRed (10,000× in water)	Biotium	41003
Ammonium persulfate (APS)	Fisher Scientific	BP179-100
Tetramethylethylenediamine (TEMED)	Thermo Scientific	PI17919
10 bp DNA ladder	ThermoFisher Scientific	SM1313
Bromophenol blue	Fisher Scientific	AAA1846918
Glycerol	Hampton Research	HR2-623
Tris base	VWR	97061-794
Acetic acid (1 M solution)	Fisher Scientific	S25840A
EDTA (0.5 M solution, pH 8)	VWR	BDH7830-1
Magnesium acetate	Fisher Scientific	BP215-500

Table S3. Reference strand displacement data from previous work corresponding to Figure 3b in the main text (*Nat. Commun.* 15: 6636, 2024, open access with permission for reuse).

	**Switchback XY**		**Duplex XZ$_L$**	
Switchback XY: Strand Z_L ratio (X-axis)	Normalized average	Std Dev	Normalized average	Std Dev
1:0	1.000	0.075	0.000	0.000
1:0.25	0.986	0.082	0.322	0.035
1:0.5	0.913	0.062	0.545	0.047
1:0.75	0.776	0.069	0.678	0.062
1:1	0.553	0.061	0.793	0.083
1:1.25	0.192	0.034	0.867	0.095
1:1.5	0.041	0.015	0.942	0.066
1:1.75	0.000	0.000	1.012	0.089
1:2	0.000	0.000	1.000	0.087

Supplementary Note 1: REAGENT PREPARATION FOR INSTRUCTOR

Apparatus required

- Weighing scale
- 500 mL Beaker
- Hotplate
- Floating rack
- 2 µl, 10 µl, 20 µl, 100 µl, and 1000 µl pipette and corresponding pipette tips
- PCR tubes or 0.5 ml or 1 ml tubes
- Tray to hold tubes
- 15 ml and 50 ml falcon tubes
- Gel loading tips (optional)
- A table-top centrifuge
- Gel electrophoresis plates, chamber, and comb (Mini-PROTEAN Tetra Vertical Electrophoresis Cell or similar)
- Power supply for gel electrophoresis (BioRad PowerPac Basic Power Supply or similar)
- GelDoc imaging station or similar (Bio-Rad Gel Doc XR+ imager)
- Orbital shaker or a rotary oscillator
- Gel analysis software such as Image Lab, Image J or similar
- Graphing software (Origin Lab or similar)

Chemicals and reagents required

- Oligonucleotides listed in Table S1.
- Reagents listed in Table S2.

Hazards

Acrylamide and TEMED are hazardous chemicals. Acrylamide is a carcinogen, and exposure can occur via inhalation (if aerosolized), ingestion, and skin absorption. Care should be taken when using acrylamide to prepare gels. TEMED is a flammable liquid and must be handled with care and used only under a chemical fume hood. Avoid contact with skin or clothing and wear personal protective equipment/face protection while handling the chemicals to prevent accidental exposure. The electrophoresis apparatus and the power supply must be handled cautiously as they pose electrical hazards. To avoid electric shocks, students should use care when plugging the gel boxes into the power supply. Exercise caution while using the gel imager by wearing appropriate skin and protection to avoid potential exposure to UV irradiation.

Preparation of reagents and solutions

Tris-Acetate-EDTA with magnesium (10× TAE-Mg^{2+}), 1 liter

Contains 400 mM Tris base (pH 8.0), 200 mM acetic acid, 20 mM EDTA, and 125 mM magnesium acetate.

48.5 g tris base
200 ml acetic acid (1 M solution)
40 ml EDTA (0.5 M solution)
26.8 g magnesium acetate
Make up to 1 liter using deionized water
Store up to 1 year at room temperature

Tris-Acetate-EDTA with magnesium (1× TAE-Mg^{2+}), 1 liter

100 ml 10× TAE-Mg^{2+} (see above recipe)
900 ml deionized water
Store up to 1 year at room temperature

10% ammonium persulfate (APS), 5 ml

0.5 g ammonium persulfate in 5 ml of deionized water (prepare fresh)

10 bp ladder (preparation for 1 lane, 10 µl)

0.5 µl 10 bp ladder
1 µl 10× TAE-Mg^{2+}
8.5 µl deionized water
Store at 4 °C and use within 24 hours.

Gel loading dye, 1 ml

500 µl glycerol
100 µl 10× TAE-Mg^{2+} (see above recipe)
400 µl deionized water
Bromophenol blue (add for visible color)

Assembly of Switchback DNA

Prepare an equimolar mixture of strands X and Y and anneal the solution in a water bath.

1. Make 20 µM dilutions for the DNA strands listed in **Table S1**.

2. Mix the 20 µM DNA strand solutions of X and Y and 10× TAE/Mg^{2+} buffer in a 0.5 ml or 1 ml tube as given below so that the final concentration of each DNA strand in the solution is 3 µM. Each student would need 120 µl of the annealed sample. The volumes of solutions given below are to

prepare switchback DNA for one student. Depending on the number of students performing the experiment, increase the volumes of the solutions proportionally, as required.

Strand X (20 μM): 18 μl
Strand Y (20 μM): 18 μl
10× TAE/Mg^{2+} buffer: 12 μl
Deionized water: 72 μl

3. Close the tubes firmly and spin down the solution with a benchtop centrifuge to get the solution to the bottom of the tubes. If preparing more volume for multiple students, split the annealed samples into aliquots of 120 μl and provide to each student.

4. Heat 400 ml deionized water to 90 °C in a 500 ml glass beaker. Seal the tubes containing the DNA samples with parafilm or teflon tape and use a floating rack to suspend the tubes from the surface of the water. Turn off the heat and let the water bath come to room temperature (20 °C) over 3-4 hours.

5. After the annealing is complete, remove the tubes from the water bath, spin down the tubes, and store the solutions at 4 °C.

6. Mix 20 μM solution of Z_L and 10× TAE/Mg^{2+} buffer as given below so that the final concentration of the DNA strand in the solution is 3 μM. Prepare this solution fresh before the reaction and let it stand at room temperature until use. Do not anneal this solution. Each student would need ~120 μl of the solution. Depending on the number of students performing the experiment, increase the volumes of the solutions proportionally, as required.

Strand Z_L (20 μM): 18 μl
10× TAE/Mg^{2+} buffer: 12 μl
Deionized water: 90 μl

Supplementary Note 2: STUDENT INSTRUCTION MANUAL

In this experiment, you will perform a strand displacement reaction on a DNA nanostructure called switchback DNA and thereby convert it into a conventional duplex DNA (Figure 1). You will incubate aliquots of switchback DNA with different concentrations of the displacement strand (Z_L). Switchback DNA contains strands X and Y. In this reaction, Z_L displaces Y and binds to X. Because Z_L is longer than Y, XZ_L duplex is larger than switchback DNA XY, and the two species appear distinctly in the gel. After incubation of the samples at room temperature, you will run the samples on a polyacrylamide gel, quantify the amount of XZ_L in each sample, and plot the yield of XZ_L against the concentration of Z_L in the sample.

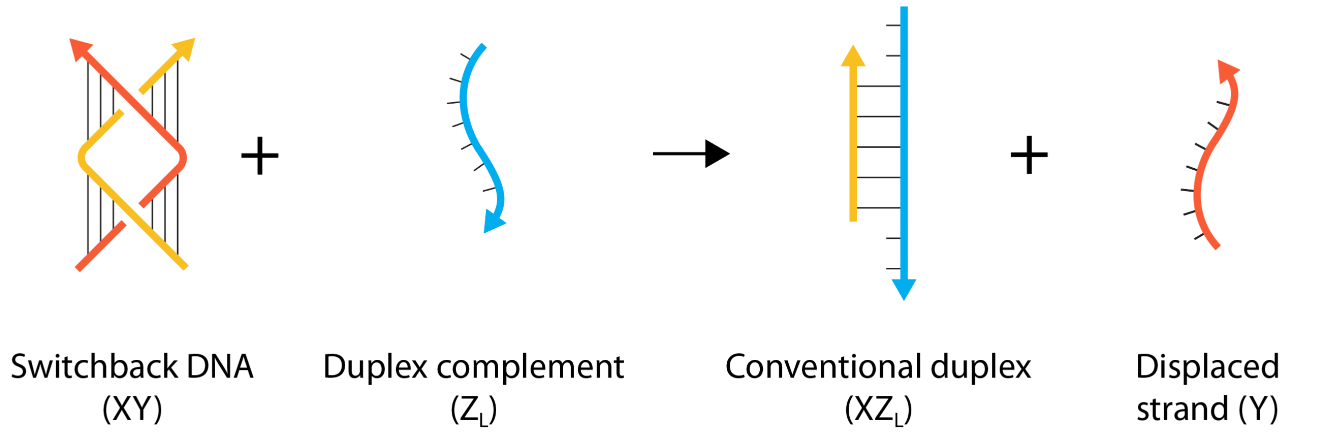

Figure 1. Scheme showing the toehold-less strand displacement of strand Y from switchback DNA XY and its conversion to a conventional duplex XZ_L.

Apparatus required

- 2 µl, 10 µl and 200 µl pipette and pipette tips
- 10 ml serological pipets
- Gel electrophoresis setup
- Orbital shaker or a rotary oscillator
- Gel imager

Chemicals and reagents required

- Annealed switchback DNA (120 µl)
- Strand Z_L solution (120 µl)
- DNA ladder (30 µl)
- 10× TAE/Mg^{2+}
- 1× TAE/Mg^{2+}
- PCR tubes or 0.5 ml tubes

- Gel Red (6 µl)
- Loading dye (30 µl)
- 40% polyacrylamide solution
- 10% ammonium persulfate (APS)
- Tetramethylethylenediamine (TEMED)

Hazards

Acrylamide and TEMED are hazardous chemicals. Acrylamide is a carcinogen, and exposure can occur via inhalation (if aerosolized), ingestion, and skin absorption. Care should be taken when using acrylamide to prepare gels. TEMED is a flammable liquid and must be handled with care and used only under a chemical fume hood. Avoid contact with skin or clothing and wear personal protective equipment/face protection while handling the chemicals to prevent accidental exposure. The electrophoresis apparatus and the power supply must be handled cautiously as they pose electrical hazards. To avoid electric shocks, students should use care when plugging the gel boxes into the power supply. Exercise caution while using the gel imager by wearing appropriate skin and protection to avoid potential exposure to UV irradiation.

Procedure

1. Prepare three non-denaturing polyacrylamide gels (20 min)

a. Mix the following to make three 18% polyacrylamide gels.

   13.5 ml 40% polyacrylamide
   3 ml 10× TAE/Mg^{2+}
   13.5 ml deionized water
   240 µl 10% APS
   12 µl TEMED

b. Set up the gel plates and chamber according to the instructor's directions.

c. Pour 10 ml of gel solution into each glass plate set up, place the combs, and allow gels to polymerize for 30-40 minutes.

d. Set up the gel chamber using 1× TAE-Mg^{2+} as the running buffer and wash the wells.

e. Keep the entire gel setup ready to be loaded when the strand displacement reaction is completed.

2. Set up the strand displacement reaction (45 min)

a. Take 27 PCR tubes and label them A1, A2, A3, B1, B2, B3, C1, C2, C3… etc. up to I1, I2, I3. A, B, C… etc. represent the different concentrations of strand Z_L you will be testing, and 1, 2, and 3 represent the three replicate experiments you will perform.

b. Pipette the switchback DNA XY, strand Z_L solution, and 1× TAE/Mg^{2+} as given below into the labeled PCR tubes to prepare samples containing mixtures of 1 µM switchback DNA (XY) and 0-2 µM strand Z_L, in triplicates.

Samples	Volume of 3 µM switchback DNA XY	Volume of 3 µM strand Z_L	Volume of 1× TAE/Mg^{2+}	Ratio
A1, A2, A3	4 µl	0 µl	8 µl	1:0
B1, B2. B3	4 µl	1 µl	7 µl	1:0.25
C1, C2. C3	4 µl	2 µl	6 µl	1:0.5
D1, D2, D3	4 µl	3 µl	5 µl	1:0.75
E1, E2, E3	4 µl	4 µl	4 µl	1:1
F1, F2, F3	4 µl	5 µl	3 µl	1:1.25
G1, G2, G3	4 µl	6 µl	2 µl	1:1.5
H1, H2, H3	4 µl	7 µl	1 µl	1:1.75
I1, I2, I3	4 µl	8 µl	0 µl	1:2

c. Mix gently by pipetting. With the instructor's guidance, spin down the tubes using a centrifuge. If needed, use empty PCR tubes to balance the centrifuge.

d. Transfer the samples to a rack and incubate the samples at room temperature for 30 min.

3. Gel electrophoresis of strand displacement reactions (65 min)

a. Set up the gel chamber using 1× TAE-Mg^{2+} as the running buffer and wash the wells.

b. Keep the entire gel setup ready to be loaded when the strand displacement reaction is completed.

c. Add 3 µl loading dye to the DNA ladder solution and keep it ready.

d. After incubating the reaction mixtures for 30 minutes, add 1 µl loading dye to all the reaction tubes. Mix the contents gently with a pipette.

e. Load 10 μl of the DNA ladder in lane 1 and 10 ul of each sample (A1-I1) into the gel, totaling 10 lanes. The order of the samples in the gel should be lane 1: ladder, lane 2: A1, lane 3: B1, lane 4: C1, etc. Load the other two gels for replicate samples (A2-I2, and A3-I3), similarly.

f. Run the gel at room temperature (20 °C) by applying 150 V for 1 hour using 1× TAE/Mg^{2+} as the running buffer.

4. Gel staining and imaging (30 min)

a. Set up three staining trays with 50 ml of deionized water in each tray. Add 2 μl of GelRed stock solution to 50 ml of deionized water to prepare the staining solution. Mix well using the pipette tip. Keep it covered with aluminum foil.

b. Transfer the gel carefully into the staining solution. For convenience, the gel can remain on one of the plates so that it is easier to move the gel.

c. Place the gel-containing trays on a shaker, cover the trays with aluminum foil, and stain the gel for 20 minutes.

d. After staining, move the gels to a tray with 50 ml water and shake them for 10 minutes to de-stain the gels.

e. Remove the gel from the de-staining tray and place it on the imaging platform (without the gel plate) on a Bio-Rad Gel Doc XR+ gel imager (or any gel imager available). Image the gel using Image Lab software package available with Bio-Rad Gel Doc XR+ (or the software available with your gel imager). Capture the gel images using different exposure times and monitor the saturation level of the bands. For analysis, use the image acquired at the highest exposure time, which did not produce saturated bands. The exposure time can be reduced to obtain gel images to avoid saturation of the bands. If saturated, the intensity values will not be accurate.

5. Gel image analysis (30-40 min)

a. Use Image Lab or the image analysis software provided with the gel imaging station (or Image J) to analyze the gel. Measure the intensity of the bands corresponding to the conventional duplex in each lane. The band in lane 2 is of switchback DNA. Conventional duplex XZ_L appears as a higher molecular weight band in all the other lanes.

b. Use the intensity of the conventional duplex band in lane 10 (Samples I1, I2, and I3) to normalize the intensities of the duplex bands in other lanes.

c. Calculate the average normalized band intensities for the triplicates and plot the band intensities of conventional duplex DNA against the concentration of strand Z_L using Origin Lab or other similar software.

d. From the plot, identify the minimum concentration of Z_L at which the switchback DNA XY is completely converted to the conventional duplex XZ_L by the strand displacement reaction.

Worksheet

1. Place your gel images.

2. Band intensity analysis.

**Sample**	**Concentration of Strand Z_L (μM)**	**Normalized band intensity (conventional duplex XZ_L)**				
		Replicate 1	Replicate 2	Replicate 3	Average	Standard deviation
A	0					
B	0.25					
C	0.5					
D	0.75					
E	1.0					
F	1.25					
G	1.5					
H	1.75					
I	2.0	1.0	1.0	1.0	1.0	0

3. The minimum concentration of Z_L required to completely convert switchback DNA to the conventional duplex XZ_L is: __________

4. Print and attach graphs below.
